# Reduction of Oxidative Stress Attenuates Lipoapoptosis Exacerbated by Hypoxia in Human Hepatocytes

**DOI:** 10.3390/ijms16023323

**Published:** 2015-02-03

**Authors:** Sang Youn Hwang, Su Jong Yu, Jeong-Hoon Lee, Hwi Young Kim, Yoon Jun Kim

**Affiliations:** 1 Department of Internal Medicine and Gastrointestinal Cancer Center, Dongnam Institute of Radiological & Medical Sciences, Busan KS012, Korea; E-Mail: mongmani@hanmail.net; 2 Department of Internal Medicine and Liver Research Institute, Seoul National University College of Medicine, Seoul KS013, Korea; E-Mails: ydoctor2@hanmail.net (S.J.Y.); pindra@empal.com (J.-H.L.); 3 Department of Internal Medicine, Seoul Metropolitan Government-Seoul National University Boramae Medical Center, Seoul KS013, Korea; E-Mail: ivtec@naver.com

**Keywords:** obstructive sleep apnea, nonalcoholic steatohepatitis, lipoapoptosis, hypoxia, endoplasmic reticulum stress, oxidative stress

## Abstract

Chronic intermittent hypoxia, a characteristic of obstructive sleep apnea (OSA), is associated with the progression of simple hepatic steatosis to necroinflammatory hepatitis. We determined whether inhibition of a hypoxia-induced signaling pathway could attenuate hypoxia-exacerbated lipoapoptosis in human hepatocytes. The human hepatocellular carcinoma cell line (HepG2) was used in this study. Palmitic acid (PA)-treated groups were used for two environmental conditions: Hypoxia (1% O_2_) and normoxia (20% O_2_). Following the treatment, the cell viability was determined by the 3,4-(5-dimethylthiazol-2-yl)-5-(3-carboxymethoxyphenyl)-2-(4-sulfophenyl)-2H-tetrazolium salt (MTS) assay, and the mechanism of lipoapoptosis was evaluated by Western blotting. Hypoxia exacerbated the suppression of hepatocyte growth induced by palmitic acid via activation of mitochondrial apoptotic pathways as a result of endoplasmic reticulum (ER) and oxidative stresses. Ammonium pyrrolidine dithiocarbamate, a scavenger of reactive oxygen species, attenuated the hypoxia-exacerbated lipoapoptosis in hepatocytes, whereas glycerol, which reduces ER stress, did not. This may have been because inhibition of oxidative stress decreases the expression of pro-apoptotic proteins, such as caspase 9 and cytochrome c. These results suggested that modulation of apoptotic signaling pathways activated by oxidative stress can aid in identifying novel therapeutic strategies for the treatment of nonalcoholic steatohepatitis (NASH) with OSA. Further *in vivo* studies are necessary to understand the pathophysiologic mechanism of NASH with OSA and to prove the therapeutic effect of the modulation of the signaling pathways.

## 1. Introduction

Nonalcoholic fatty liver disease (NAFLD) is characterized by hepatic steatosis along with inflammation in individuals without significant alcohol consumption. Nonalcoholic steatohepatitis (NASH) is a relatively aggressive form of NAFLD, because it may progress to cirrhosis and even to hepatocellular carcinoma [1,2]. The pathogenesis of NASH is multifactorial and is related to systemic metabolic disease associated with insulin resistance, such as obesity and hyperlipidemia, which increase circulating levels of free fatty acids (FFA) [3]. Saturated FFAs are hepatotoxic, in part by promoting lipoapoptosis, which is a key histologic feature of NASH [4]. It is suggested that lipoapoptosis of hepatocyte and serum biomarkers of this pathologic process correlate with NASH severity [5]. Obstructive sleep apnea (OSA) is also a common condition in obese patients, characterized by recurrent collapse of the upper airway during sleep. Some authors suggested that chronic intermittent hypoxia (CIH), characteristic of OSA, may play a causative role in the progression of simple hepatic steatosis to a necroinflammatory hepatitis, even to fibrosis through enhancement of apoptosis [6,7,8,9]. However, the mechanism by which hypoxia increases lipoapoptosis is still poorly understood.

Thus, in this study, we examine the following: (i) What is the main apoptotic pathway that hypoxia enhanced? (ii) What is the mechanism by which hypoxia activated apoptotic signaling? (iii) Did the modulation of that mechanism attenuate lipoapoptosis enhanced by hypoxia in a hepatocyte cell line?

## 2. Results

### 2.1. Hypoxia Exacerbates Growth Suppression in Hepatocytes Treated with Palmitic Acid (PA)

**Figure 1:**
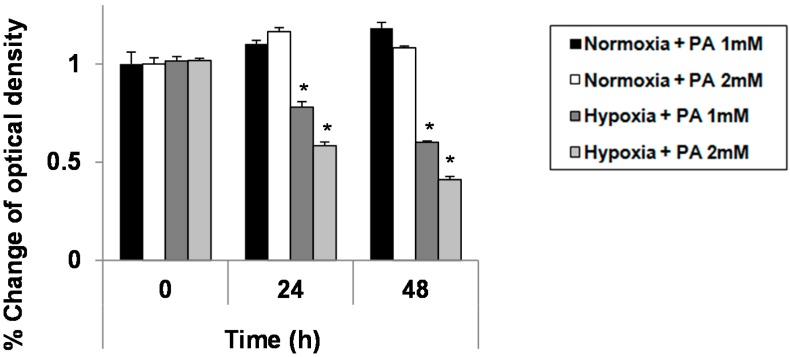
Enhanced palmitic acid (PA)-induced growth suppression in hypoxic hepatocytes. HepG2 cells grown under either standard culture conditions (20% O_2_ and 5% CO_2_; at 37 °C) or hypoxic culture conditions (1% O_2_, 5% CO_2_ and 94% N_2_; at 37 °C) were treated with 1.0 mM of PA. At each indicated time point, a 3,4-(5-dimethylthiazol-2-yl)-5-(3-carboxymethoxyphenyl)-2-(4-sulfophenyl)-2H-tetrazolium salt (MTS) assay was performed according to the manufacturer’s instructions. Data were expressed as the mean ± standard deviation of the percent change of optical densities compared to Time 0 h. * *p* < 0.05.

To investigate the effect of hypoxia on cell growth or the viability of HepG2 treated with palmitic acid (PA), HepG2 cells was treated with two different doses of PA (1 and 2 mM) and two different conditions (normoxia and hypoxia) for 24 and 30 h, and the cell viability was measured by the MTT assay. Growth suppression was significantly greater in hypoxic cells than in normoxic cells in a dose-dependent manner (*p* < 0.05; Figure 1). This finding implies that hypoxia exacerbates PA-induced growth suppression.

### 2.2. Hypoxia Exacerbates Lipoapoptosis Occurring via Mitochondrial Pathways

**Figure 2:**
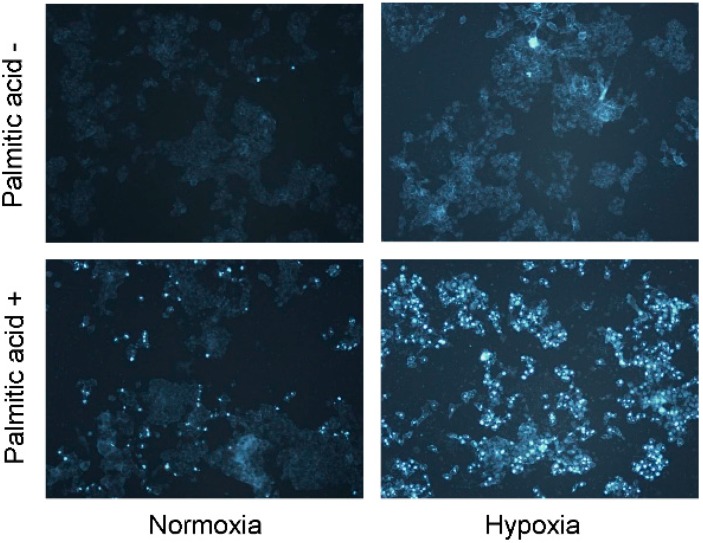
HepG2 cells were cultured with PA (1 mM) under either standard or hypoxic culture conditions for 24 h. Apoptosis was assessed by 4',6-diamidino-2-phenylindole dihydrochloride (DAPI) staining. Original magnification, ×200.

**Figure 3:**
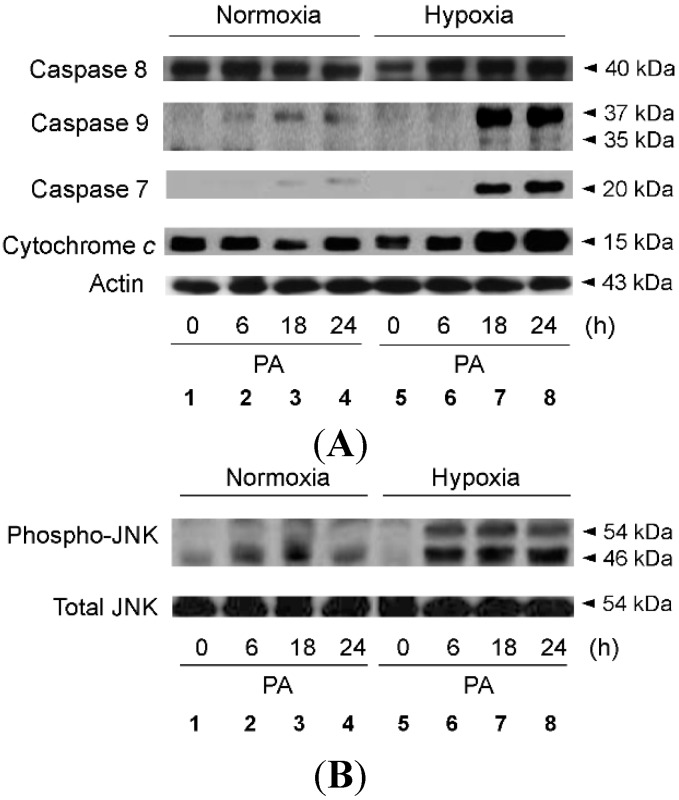
Enhanced PA-induced activation of caspase 9 and JNK in hypoxic hepatocytes. HepG2 cells were cultured with PA (1 mM) under either standard or hypoxic culture conditions for the indicated time periods. Immunoblot analysis was performed using anti-caspase 7, anti-caspase 8, anti-caspase 9 and anti-actin antibody (**A**); and anti-phospho-JNK and anti-JNK antibody (**B**).

We attempted to determine whether hypoxia-exacerbated growth suppression was due to induction of apoptotic cell death, and as shown in Figure 2, we found that PA-induced apoptosis was increased significantly in hypoxic hepatocytes, as compared with normoxic cells. We further explored whether PA-induced signaling pathways for apoptosis were more active in hypoxic cells. As shown in Figure 3A, the apoptotic pathways activated by PA were primarily mitochondrial, such as those leading to activation of caspase-9, and these pathways were more active in hypoxic cells than in normoxic cells (Figure 3B; Lanes 3 and 4 *vs.* Lanes 7 and 8). We also found that PA activated c-Jun *N*-terminal kinases (JNKs), one of the many mitogen-activated protein kinases, which were likewise more active in hypoxic cells than in normoxic cells (Figure 3B; Lanes 3 and 4 *vs.* Lanes 7 and 8). Because JNK activation has previously been reported to trigger the mitochondrial apoptotic pathway, it is probable that the greater apoptotic activity in hypoxic cells was due to greater JNK activity following PA treatment.

**Figure 4:**
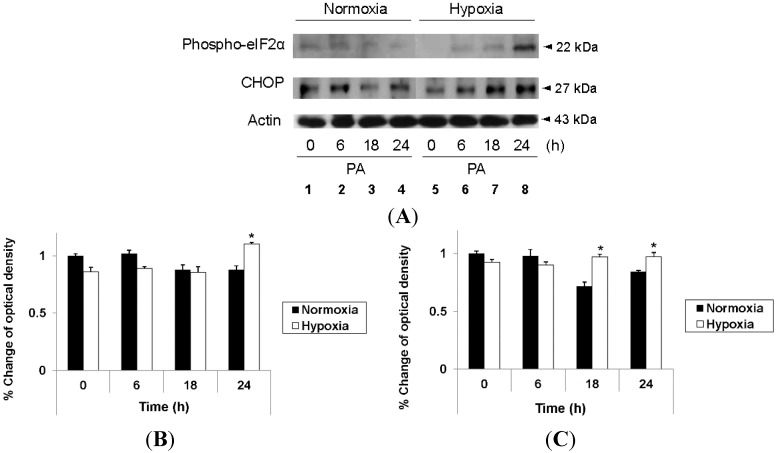
Enhanced PA-induced ER stress in hypoxic hepatocytes. HepG2 cells were cultured with PA (1 mM) under either standard or hypoxic culture conditions for the indicated time periods. Immunoblot analysis was performed using anti-phospho-eIF2a, anti-CHOP and anti-actin antibody (**A**); Densitometric evaluation was performed for anti-phospho-eIF2α (**B**); and anti-CHOP (**C**). Data are expressed as the mean ± standard deviation of the percent change of optical densities compared to Time 0 h. * *p* < 0.05.

### 2.3. Hypoxia Exacerbates Endoplasmic Reticulum Stress Leading to Activation of Mitochondrial Apoptotic Pathways

As endoplasmic reticulum (ER) stress can lead to JNK activation, we attempted to determine if PA induces ER stress in hepatocytes. As shown in Figure 4A, PA increased the expression of CCAAT/enhancer-binding protein homologous protein (CHOP) and phosphorylation of eukaryotic initiation factor 2α (eIF2α), which are markers of ER stress, with both being significantly higher in hypoxic cells than in normoxic cells (Lanes 3 and 4 *vs.* Lanes 7 and 8). These findings were also consistent in densitometric evaluation (Figure 4B,C). These findings indicate that PA induces ER stress, thereby leading to JNK activation and apoptosis, and that this signaling cascade is more active in hypoxic hepatocytes than in normoxic ones.

**Figure 5:**
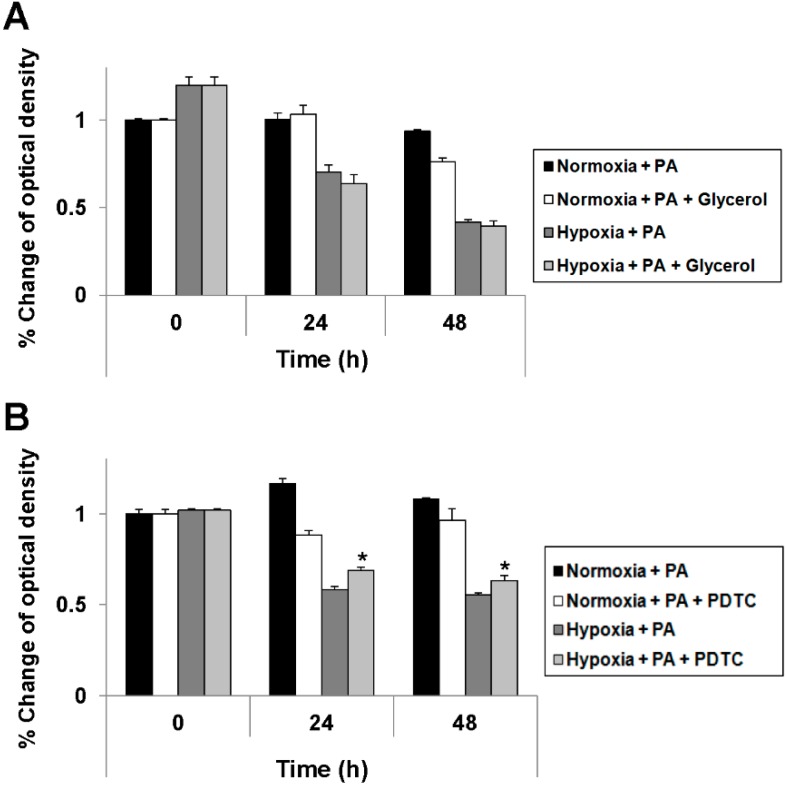
Attenuation of hypoxia-exacerbated lipoapoptosis by pyrrolidine dithiocarbamate (PDTC). HepG2 cells were cultured in the absence or presence of glycerol (1%) under either standard or hypoxic culture conditions (**A**); HepG2 cells were cultured in the absence or presence of PDTC (0.6 mM) under either standard or hypoxic culture conditions (**B**). At each indicated time point, an MTS assay was performed according to the manufacturer’s instructions. Data are expressed as the mean ± standard deviation of the percent change of optical densities compared to Time 0 h. * *p* < 0.05.

### 2.4. Reduction of Oxidative Stress Attenuates Exacerbation of Lipoapoptosis by Hypoxia

**Figure 6:**
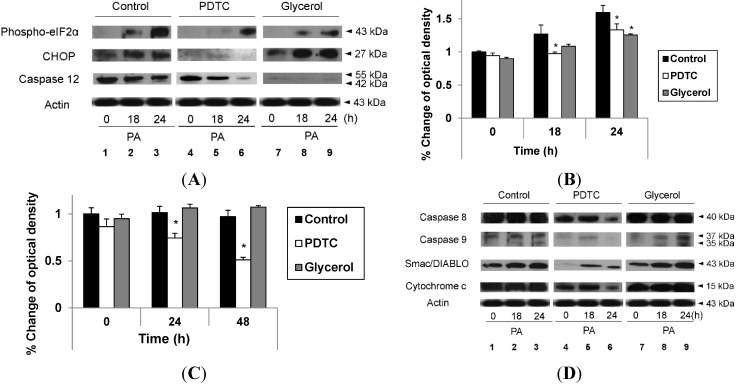
Attenuation of hypoxia-exacerbated ER stress by both PDTC and glycerol and attenuation of hypoxia-exacerbated lipoapoptosis by PDTC. HepG2 cells were cultured with PA (1 mM) under hypoxic culture conditions and were treated in three different manners for the indicated time periods: no treatment (control), PDTC (0.6 mM) and glycerol (1%). Immunoblot analysis was performed using anti-phospho-eIF2α, anti-CHOP, anti-caspase 12 and anti-actin antibody (**A**); Densitometric evaluation was performed for anti-phospho-eIF2α (**B**); and anti-CHOP (**C**); Immunoblot analysis was also performed using anti-caspase 8, anti-caspase 9, anti-Smac/DIABLO, anti-cytochrome c and anti-actin antibody (**D**). Data are expressed as the mean ± standard deviation of the percent change of optical densities compared to Time 0 h. * *p* < 0.05.

We attempted to determine whether the reduction of ER stress would attenuate the exacerbation of PA-induced cell death by hypoxia. For this purpose, we treated hepatocytes with glycerol, a strong suppressor of ER stress; however, as shown in Figure 5A, there was no effect. Accordingly, we suspected the mechanism of exacerbation to be upstream of the effects on the ER. As oxidative stress can trigger the mitochondrial apoptotic pathway and hepatic hypoxia or hypoperfusion can induce the production of reactive oxygen species (ROS), which cause oxidative stress, we attempted to determine whether reducing oxidative stress would attenuate the exacerbation of PA-induced cell death by hypoxia. For this purpose, we treated hepatocytes with ammonium pyrrolidine dithiocarbamate (PDTC), an ROS scavenger. As shown in Figure 5B, PDTC successfully attenuated the exacerbation of PA-induced cell death by hypoxia. Finally, we attempted to determine if the reduction in cell death was due to the attenuation of the apoptotic signaling pathway. Although both PDTC and glycerol attenuated signaling pathways related to ER stress (Figure 6A, Lane 3 *vs.* Lane 6 *vs.* Lane 9), only PDTC attenuated apoptotic signaling (Figure 6D, Lane 3 *vs.* Lane 6 *vs.* Lane 9). These findings were also consistent in the densitometric evaluation (Figure 6B,C) for phosphor-eIF2α and CHOP. These findings indicate that lipoapoptosis exacerbated by hypoxia may be attenuated by the reduction of oxidative stress, but not by reduction of ER stress.

## 3. Discussion

Our key findings were: (i) That hypoxia exacerbated PA-induced growth suppression in hepatocytes by activation of mitochondrial apoptotic pathways; and (ii) That although this exacerbation occurred via both ER stress and oxidative stress, only the reduction of the latter attenuated lipoapoptosis.

**Figure 7:**
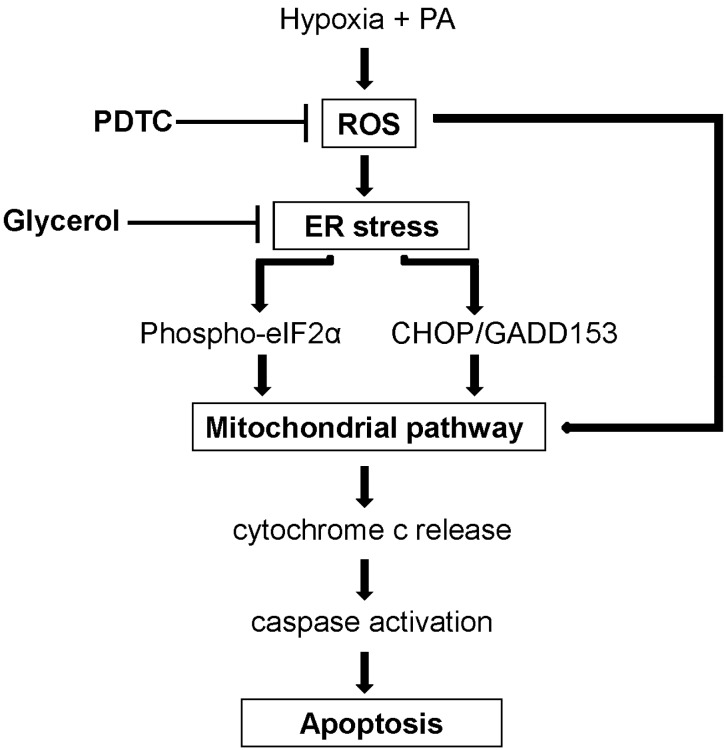
Proposed pathogenic model for the signal transduction pathway involved in hypoxia-aggravated lipoapoptosis.

In this study, we use palmitic acid for inducing lipoapoptosis, because saturated fatty acids, such as palmitic acid, resulted in increased hepatocyte apoptosis and impaired insulin singling, whereas oleic acid is more steatogenic [10]. Then, we showed that hypoxia exacerbates lipoapoptosis via mitochondrial apoptotic signaling pathways by causing greater ER and oxidative stresses. ER stress is caused by accumulation of misfolded proteins in the ER, which can result from various insults. Under ER stress, eIF2α is phosphorylated to activate the unfolded protein response (UPR), which aims to attenuate ER stress and, accordingly, avoid cell damage [11,12]. However, in spite of the UPR, prolonged ER stress mediates apoptosis through activation of JNKs, caspase 12 and CHOP, as observed in the current study [13,14,15,16,17,18]. Oxidative stress due to increased generation of ROS and decreased antioxidant defenses is also important in the pathogenesis of NASH. Exacerbated mitochondrial and microsomal β-oxidation of fatty acids, induction of cytochrome P450 2E1 (CYP2E1) and leukocyte infiltration can all lead to oxidative stress, which can trigger the mitochondrial apoptotic pathway via activation of pro-apoptotic members (BAX, BAK) that oligomerize on the outer mitochondrial membrane and cause mitochondrial dysfunction [19,20,21]. As hypoxia is well known to induce both ER and oxidative stresses [22,23], these stresses may have a synergistic effect, resulting in exacerbation and prolongation of both and leading to cellular apoptosis. Indeed, we found that although ER stress was attenuated by both glycerol and PDTC, only inhibition of oxidative stress successfully attenuated the exacerbation of PA-induced apoptosis by hypoxia, probably because apoptosis was mainly induced by membrane destruction as a result of oxidative stress generated upstream of the ER [24]. We proposed the pathogenic model of hypoxia-aggravated lipoapoptosis based on the findings of our study (Figure 7).

Previous studies have estimated the prevalence of NAFLD in obese persons to be up to 75% and that of NASH in this population to be up to 35%. NASH progresses to cirrhosis in 15%–25% of patients, and over a 10-year period, 30%–40% of cirrhotic NASH patients will die of liver-related causes [1,3]. Although the pathophysiology of NASH still has not been completely determined, the so-called “multiple parallel hits” theory is now generally accepted [25]. The hypothesis emphasizes that variable inflammatory mediators derived from the gut and adipose tissue could be attributed to the cascade of inflammation, fibrosis, *etc.* Additionally to increased lipid storage, cytokine synthesis may induce ER stress, which is considered as an important player in driving hepatocyte apoptosis, resulting in progression of NASH. Moreover, several reports suggested that NAFLD precedes and is a risk factor for the future development of diabetes and metabolic syndrome by insulin resistance, considered as a major player. Insulin resistance could trigger ER stress, the production of ROS, mitochondrial dysfunction, *etc*. [26].

OSA affects 1%–4% of the general population, but the prevalence rises to 40%–60% in obese individuals. OSA can lead to CIH through repetitive pharyngeal collapse during sleep and has been associated with an increased risk of cardiovascular and metabolic disorders, such as hypertension, type 2 diabetes and dyslipidemia [27]. Several studies have suggested OSA to be associated with greater insulin resistance, greater degrees of hepatic steatosis and fibrosis and elevated aminotransferase levels. It has also been suggested that hypoxia associated with OSA can act as the “second hit” in the pathogenesis of NASH [7,8,9,28,29]. One meta-analysis reported that the odds ratio (OR) of OSA for NASH was 2.37 (1.59–3.51) [30], and another recent study reported that the severity and presence of OSA had a 4.89 (3.08–5.98) OR for the presence of NASH in pediatric NAFLD [31]. Another study suggested that hypoxia inducible factor-1 α (HIF-1α) had an important role in the progression of NASH. CIH induces activation of HIF-1α via the activation of nuclear factor κB (NF-κB) pathways. HIF-1α is a master regulator of oxygen homeostasis, and activation of HIF-1α is associated with the sympathetic response, increased triglyceride levels and insulin resistance, linking the hypoxic and proinflammatory response pathways [32]. Moreover, a recent study reported that HIF-1α showed a causative role of liver injury in alcoholic fatty liver [33]. In contrast, another study reported that upregulation of HIF-1α reduces lipoapoptosis in NAFLD; however, this protective effect of HIF-1α disappeared with persistent lipid exposure [34]. Anyhow, there have been few studies of the pathophysiological mechanisms of the putative relationship between OSA and NASH, particularly with regard to how hypoxia might be associated with lipoapoptosis, which is NASH’s key morphological and pathological feature. Therefore, we tried to concentrate our efforts on investigating the main lipoapoptotic pathway enhanced by hypoxia and modulating the revealed signaling pathways.

The limitations of this study include that the experiments were performed using only one cell line and that no *in vivo* experiments were performed. Thus, although PDTC attenuated the exacerbation of PA-induced lipoapoptosis by hypoxia, our data were insufficient to determine whether it would be an effective treatment. In addition, we did not attempt a detailed investigation of why the reduction of oxidative stress attenuated the exacerbation of lipoapoptosis by hypoxia, whereas the reduction of ER stress did not. Further studies involving other hepatocyte lines and *in vivo* experiments are required to completely elucidate the mechanism by which hypoxia exacerbates lipoapoptosis in hepatocytes.

In conclusion, our current study demonstrates that PA-induced apoptosis is more widespread in hypoxic hepatocytes than in normoxic ones as a result of the activation of mitochondrial apoptotic pathways due to hypoxia-related ER and oxidative stresses. The reduction of oxidative stress attenuates this hypoxia-exacerbated apoptosis by hypoxia, whereas the reduction of ER stress does not. Thus, the modulation of the signaling pathways associated with oxidative stress may be useful in the treatment of hepatic steatosis patients with OSA and may prevent NASH and fibrosis as a result of hypoxia.

## 4. Materials and Methods

### 4.1. Cell Lines and Culture

The human HCC cell line that was used in this study is HepG2, a well-differentiated human hepatoblastoma cell line that retains many characteristics of normal differentiated quiescent hepatocytes. Cells were grown in Dulbecco’s modified Eagle’s medium (DMEM, Welgene, Daegu, Korea) supplemented with 10% fetal bovine serum (FBS; HyClone, Logan, UT, USA), 100,000 U/L penicillin (Gibco, Carlsbad, CA, USA) and 100 mg/L streptomycin (Gibco). Cells were incubated either under standard culture conditions (20% O_2_ and 5% CO_2_, at 37 °C) or under hypoxic culture conditions (1% O_2_ and 5% CO_2_, at 37 °C). To avoid confounding factors related to serum-induced signaling, cells were serum starved overnight for all of the experiments.

### 4.2. Cell Proliferation Analysis

Cell growth was measured colorimetrically using the CellTiter 96^®^ AQueous One Solution Cell Proliferation Assay (Promega Corporation, Madison, WI, USA), which is based on the cellular conversion of 3,4-(5-dimethylthiazol-2-yl)-5-(3-carboxymethoxyphenyl)-2-(4-sulfophenyl)-2H-tetrazolium salt (MTS, Promega Corp., Madison, WI, USA) into soluble formazan by dehydrogenase enzymes produced only in metabolically active, proliferating cells. Following each treatment, 20 μL of CellTiter 96^®^ AQueous One Solution reagent was added into each well of a 96-well plate. After 1 h of incubation, the absorbance for each well at a 490 nm wavelength was recorded using an ELISA plate reader (Molecular Devices, Sunnyvale, CA, USA).

### 4.3. Reagents

Palmitic acid (PA) was obtained from Sigma (St. Louis, MO, USA). Glycerol was obtained from Wako Pure Chemical Industries Ltd. (Osaka, Japan). Ammonium pyrrolidine dithiocarbamate (PDTC) was obtained from Sigma.

### 4.4. Apoptosis Analysis

Apoptosis was assessed by examining apoptosis-associated nuclear changes (*i.e*., chromatic condensation and nuclear fragmentation) using DNA binding dye, 4',6-diamidino-2-phenylindole dihydrochloride (DAPI) and fluorescence microscopy (Carl Zeiss, Jena, Germany).

### 4.5. Immunoblot Analysis

Cells were lysed for 20 min on ice with lysis buffer (50 mM Tris-HCl (pH 7.4); 1% Nonidet P-40; 0.25% sodium deoxycholate; 150 mM NaCl; 1 mM EDTA; 1 mM PMSF; 1 μg/mL aprotinin, leupeptin and pepstatin; 1 mM Na_3_VO_4_; and 1 mM NaF) and centrifuged at 14,000× *g* for 10 min at 4 °C. Samples were resolved with 10% or 12% sodium dodecyl sulfate polyacrylamide gel electrophoresis, transferred to nitrocellulose membranes and blotted using appropriate primary antibodies with peroxidase-conjugated secondary antibodies (Biosource International, Camarillo, CA, USA). Bound antibodies were visualized using a enhanced chemiluminescent substrate (ECL; Amersham, Arlington Heights, IL, USA) and exposed to film (X-Omat; Kodak, Hannover, Germany). The primary antibodies used were: mouse anti-phospho-c-Jun *N*-terminal kinase (JNK), mouse anti-JNK and rabbit anti-phospho-eukaryotic initiation factor 2α (eIF2α), purchased from Cell Signaling Technology (Beverly, MA, USA); rabbit anti-caspase 8 and rabbit anti-caspase 9, from Pharmingen (San Diego, CA, USA); and Smac/DIABLO, cytochrome c and CHOP from Santa Cruz Biotechnology Inc. (Santa Cruz, CA, USA). The cleaved form of caspases was analyzed and expressed in all of the experiment.

### 4.6. Data Analysis

All experimental results represent at least 3 independent experiments using cells from a minimum of 3 separate isolations and are expressed as the means ± standard deviation. Comparisons between groups used a Mann–Whitney *U*-test. All analyses used SPSS version 12.0 (SPSS Inc., Chicago, IL, USA). *p* < 0.05 was considered significant.
